# Branched tyramides from males of the harvester ant, *Pogonomyrmex badius*

**DOI:** 10.1007/s00114-023-01885-2

**Published:** 2023-12-07

**Authors:** Tappey H. Jones, Satya P. Chinta, Robert K. Vander Meer, Kaitie C. Cartwright

**Affiliations:** 1 Department of Chemistry, Virginia Military Institute, Lexington, VA 24450 USA; 2 Foresight Science & Technology, Hopkinton, MA 01748 USA; 3 USDA/ARS, CMAVE, 1600 SW 23rd Drive, Gainesville, FL 32608 USA

**Keywords:** Reproductive inhibition, Activation, Colony success, Species separation

## Abstract

Tyramides are produced in microgram quantities by males of species in the large Myrmicine ant sub-family (> 7000 species). Tyramides are transferred to female sexuals during mating where a specific female sexual evolved enzyme hydrolyzes the tyramides to the biogenic amine, tyramine. Tyramine is a ligand for receptors that rapidly activate reproductive development in the newly mated queen—previously reproductively inhibited by the mother queen. Without this elaborate biogenic amine precursor and co-evolved female sexual derived tyramide hydrolase, the defenseless newly mated queen’s worker production would be delayed by up to 6 days, which could be lethal to the new queen. This is one of possibly several ant species separation mechanisms evolved to maintain species integrity. Here we report two methyl-branched tyramides from harvester ant, *Pogonomyrmex badius,* males, including one highly branched tyramide not previously reported.

## Introduction

Tyramides were first identified from males of several ant species by Jones et al. (2010) and Adams et al. (2010), then expanded to males of the black imported fire ant, *Solenopsis richteri* Forel (Chen and Grodowitz 2017). A breakthrough came when tyramides produced by red imported fire ant males (*Solenopsis invicta* Buren) were identified and their function elucidated (Vander Meer et al. 2021). The colony queen releases primer pheromones that inhibit reproductive development in her sexual daughters to prevent within colony competition. Primer pheromone effects can last up to 6 days (Burns et al. 2005). However, her sexual daughters need to overcome the queen’s primer pheromone immediately after their mating flight and mating. This is accomplished through male-derived tyramides. These compounds are produced in the male reproductive system and are transferred to the winged female sexuals at mating. The female sexuals produce specific tyramide hydrolases that convert tyramides to the biogenic amine, tyramine. Tyramine is then transported into the newly mated queen’s hemolymph, where it is a ligand for tyramine receptors that trigger wing loss, ovariole development, and other reproductive development actions in newly mated queens. While the fire ant, *S. invicta*, serves as a specific model, it appears that tyramides are associated with males within the large Myrmicinae ant sub-family (> 7000 species). Luo et al (2022) expanded the scope of tyramide structures through a survey of male tyramides from 15 fungus growing ant species, including methyl branched tyramides. The blend of male tyramides and the specific hydrolases produced by female sexuals from each species are expected to add another layer of mating species specificity to what is already known. Here we describe two methyl-branched tyramides from males of the harvester ant, *Pogonomyrmex badius*, one known and the other a highly methyl branched tyramide described for the first time.

## Methods

### Pogonomyrmex badius males

Male *P. badius* were collected from 1) mating flights at Watermelon Pond, a part of the Goethe State Forest located in Northwest Florida. The terrain was sandy scrub pine. During mating activity sexual females from a colony move to the surface, presumably release sex pheromones, and wait for males from other colonies to fly to them (Harmon 1993 and Rheindt et al. 2004). Males were aspirated into a container, transferred to vials, then cooled in an insulated container with ice. 2) Males were also collected from excavated colonies in the same general location. In the laboratory, 7-ml scintillation vials (DWK Life Sciences, Millville, NJ, USA) were used to segregate the collected males into single males and groups of 3. Then, enough methanol (Fisher Scientific, Inc.) was added to each vial to cover the *P. badius* males. Tyramides were passively extracted unless otherwise indicated. The male samples were stored in a freezer until use.

### Chemical analyses

The male extracts were subjected to gas chromatography–mass spectrometry (GC–MS). Analyses were carried out in electron impact (EI) mode using a Shimadzu QP-2020 GC–MS equipped with an RTX-5, 30 m × 0.25 mm i.d. column. The instrument was programmed from 60 to 250 °C at 10 °C/min and held at that temperature for 20 min.

### NMR

NMR spectra were obtained in CDCl_3_ using a JEOL 400 MHz NMR spectrometer. Spectra were referenced to residual protio chloroform solvent signals.

## Results

*Pogonomyrmex badius* tyramide 1**:** 2-methylbutanoyl tyramide, formally: N-[2-(4-hydroxyphenyl)ethyl]-2-methylbutanamide was identified by comparison with a synthetic sample (Luo et al. 2022). This component comprises about 2% of the two identified tyramides. Its retention index is 2052.

**Fig. 1:**
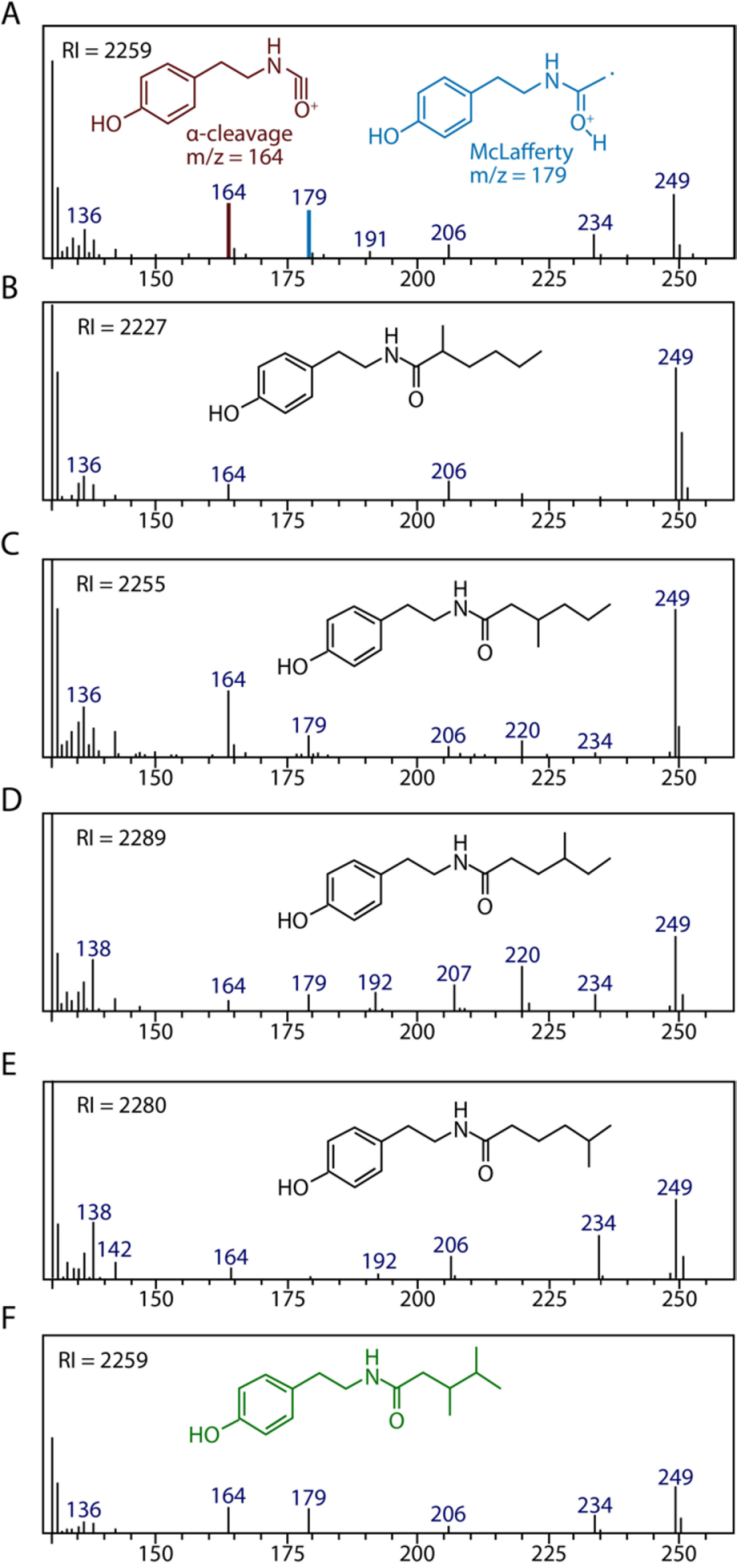
Expanded mass spectral fragmentation patterns from *m*/*z* = 130 to *m*/*z* = 250. Spectra A and F are *P. badius* tyramide-2. Spectra B, C, D, and E are the 4 possible mono methyl branched hexanoyl tyramides. The fragments at *m*/*z* = 164 and *m*/z = 179 are due to the indicated α-cleavage and McLafferty rearrangement, respectively (**A**). The retention index (RI) is shown (upper left corner) for each spectrum

*Pogonomyrmex. badius* Tyramide 2**:** 3,4-dimethylpentanoyl tyramide, formally: N-[2-(4-hydroxyphenyl)ethyl]-3,4-dimethylpentanamide. This new tyramide is described here for the first time. It represents about 98% of the two tyramides. Its retention index is 2259; see Fig. 1A (natural) and Fig. 1F (synthetic).

Methylhexanoyl tyramide standards. Four isomeric methylhexanoyl tyramides were prepared as previously described (Luo et al. 2022): 5-methylhexanoyl- (Fig. 1B); 4-methyhexanoyl- (Fig. 1C); 3-methylhexanoyl- (Fig. 1D); and 2-methylhexanoyl-tyramide (Fig. 1E) (retention Indices are in upper left corner of each spectrum).

3,4-dimethylpentanoic acid. This compound is commercially available and there is a published synthesis (Sheehan and Ledis 1973); however, we used a simple unambiguous 3-step synthesis of the racemic product. The condensation of 3-methyl-2-butanone with triethylphosphonoacetate under standard Horner-Wadsworth-Emmons conditions provided an isomeric mixture of ethyl-3,4-dimethyl-2-pentenoate which was immediately hydrogenated in the presence of 10% Pd/C, and the resulting ethyl-3,4-dimethylpentanoate was converted to 3,4-dimethylpentanoic acid by saponification with aqueous KOH and subsequent acidification.

3,4-dimethylpentanoyl tyramide. 3,4-dimethylpentanoic acid was converted into its acid chloride, then condensed with tyramine (see Luo et al. 2022) to yield the corresponding 3,4-dimethylpentanoyl tyramide. Its mass spectrum and gas chromatographic retention time matched those of the *P. badius* tyramide 2 with a retention index of 2259.

NMR: 3,4-dimethylpentanoyl tyramide. ^*1*^*H NMR* (400 MHz, CHLOROFORM-*D*) δ 6.99 (d, *J* = 8.5 Hz, 2H), 6.79 (d, *J* = 8.6 Hz, 2H), 5.62 (broad s, 1H), 3.48 (q, *J* = 6.4 Hz, 2H), 2.71 (t, *J* = 7.0 Hz, 2H), 2.20 (dd, *J* = 12.4, 3.6 Hz, 1H), 1.865 (m, 1H) 1.82 (dd, J = 12.4, 8.5Hz, 1H), 1.59 – 1.50 (m, 1H), 0.83 (d, *J* = 6.8 Hz, 3H), 0.80 (d, *J* = 6.8 Hz, 3H), 0.78 (d, *J* = 6.8 Hz, 3H).

^*13*^*C{*^*1*^*H} NMR* (101 MHz, CHLOROFORM-*D*) δ 173.9, 155.3, 130.1, 129.9(2C), 115.8(2C), 41.9, 41.0, 36.4, 34.9, 32.1, 20.0, 18.5, 15.6.

EIMS *m*/*z* 249[M^+^](1), 234(0.3), 206(0.1), 179(1), 164(1), 130(15), 121(10), 120(100), 107(5), 95(4), 77(2), 71(1), 43(8).

The mass spectra of tyramides are dominated by a base peak at *m*/*z* = 120, and most of the other fragment ions have a much lower relative intensity, e.g., benzylic cleavage at *m*/*z* = 107 (< 5%) and usually there is a weak parent ion. Tyramide 2 from *Pogonomyrmex badius* similarly has a base peak at *m*/*z* = 120 and shows a weak parent at *m*/*z* = 249 (< 1%). Treatment of tyramide-2 with NaBH_4_ did not change the structure and, therefore, ruled out the presence of a ketotyramide. Tyramide-2 is isomeric with n-heptanoyl tyramide (MW = 249), but not identical. The alkyl chain is C_6_H_13_. The retention time of tyramide 2 suggested a methyl branched hexanoyl tyramine, parent *m*/*z* = 249; however, none of the four possible methyl- isomers (Fig. 1 B–E, Luo et al. 2022) matched the retention index or fragmentation pattern of *P. badius* tyramide-2 (Fig. 1A and 1F).

Expansion of the mass fragmentation pattern above *m*/*z* = 130 revealed smaller fragments. For example, *m*/*z* = 164 results from α-cleavage next to the carbonyl group and *m*/*z* = 179 results from a McLafferty rearrangement where there is no substituent on the α-carbon. Additionally, fragments at *m*/*z* = 234 (M-CH_3_) and 206 (M-C_3_H_7_) were observed. However, fragments at 220 (M-ethyl) and *m*/*z* 192 (M-C_4_H_9_) were missing, suggesting that sidechain positions 3 and 4 have methyl branching. Considering these data, a sample of 3,4-dimethylpentanoic acid (Sheehan and Ledis 1973) was prepared and converted into its acid chloride and then condensed with tyramine (Luo et al. 2022) to provide 3,4-dimethylpentanoyl tyramide whose mass spectra and gas chromatographic retention index matched that of *P. badius* tyramide-2 (Fig. 1A and 1F). The NMR analyses were carried out on synthetic *P. badius* tyramide-2 only to confirm its structure. NMR data were not obtained for natural *P. badius*-2 due to the small amount available.

## Discussion

Biogenic amines, tyramine and octopamine, are considered the epinephrine and nor-epinephrine of the insect world (Roeder 2005). The biogenic amines are ligands that activate their corresponding GPCRs (G-protein-coupled receptors), e.g., when tyramine binds with its specific GPCR a physiologically critical biochemical sequence is initiated. Interestingly, the mechanism that evolved to appropriately stop this biochemical reaction is illustrated in the ant, *Formica japonica*, and involves acetylation of tyramine to acetyl tyramide (Aonuma and Watanabe 2012). The same mechanism was reported for octopamine where deactivation produced acetyl octopamide. This discovery provides a precedence for the biochemical machinery needed for the biosynthesis of tyramides in male ants. We postulate that over evolutionary time, there was pressure for queens to inhibit reproductive development in their sexual daughters through primer pheromones. It takes time for primer pheromones to exert their physiological effects and conversely, it takes up to 6 days for the inhibited sexual daughters to develop reproductively when out of the influence of the mother queen (Burns et al. 2005). We discovered that immediately after mating reproductive development commences (Vander Meer et al. 2021). Further we linked this rapid development to the newly mated female sexual hydrolysis of male-derived tyramides to the biogenic amine tyramine (Vander Meer et al. 2021).

In ants, species-specific lineages can be maintained through multiple avenues, e.g., distance, mating flight timing/flight altitude/visual landmarks/male leks, or female sex pheromones. Here we suggest that the species specificity of tyramides and their hydrolases may also contribute to maintenance of species separation. To date, only males from species of the largest ant sub-family, Myrmicinae (7102 species, AntWeb 2023) produce tyramides. If a mistake is made and the tyramides are not transferred to the female sexual at mating or if a female sexual mates with a male from a different species, rapid reproductive development will not occur, and the potential new colony will likely not survive due to slow development and the high risk from opportunistic predation.

Remarkably, there is an example illustrating how viable hybrids are possible. *Solenopsis richteri* from southern Argentina/Uruguay is geographically separated from *Solenopsis invicta* from northern Argentina/Brazil. However, *Solenopsis richteri* and *S. invicta* were accidentally imported into Mobile, AL, USA, around 1918 and early 1930s, respectively (Wilson 1951). Males of the two species have the same tyramide composition (acetyl and hexanoyl tyramide). While it is likely that their respective female sexuals do not have identical tyramide hydrolases, both enzymes evolved to act on the same two tyramides. Therefore, the two species could hybridize and maintain the tyramine jump-start in reproductive development. Indeed, viable hybridization was discovered for the two imported *Solenopsis* species, first via chemical characters (Vander Meer et al. 1985), then via genetic markers (Ross et al. 1987).

While *P. badius* is a member of the Myrmicinae sub-family and males produce tyramides, it differs in its mating strategy from most other members of this sub-family. Instead of female sexuals flying into a large male lek to be mated, as with the fire ant, *P. badius* female sexuals rest on top of their colony and attract males to them for mating. The end result appears to be the same; mating males transfer tyramides to the female sexuals that then hydrolyze them to tyramine. It is clear that the intricacies of this complex co-evolved enzyme/ligand system will continue to be revealed as additional discoveries are made.

## Data Availability

The data generated and ants collected during and/or analyzed during the current study are available from the corresponding author on reasonable request.

## Code availability

N.A.
